# Genome-Wide Comparison and Functional Characterization of *HMGR* Gene Family Associated with Shikonin Biosynthesis in *Lithospermum erythrorhizon*

**DOI:** 10.3390/ijms241512532

**Published:** 2023-08-07

**Authors:** Xuan Wang, Changyi Wang, Minkai Yang, Wencai Jie, Aliya Fazal, Jiangyan Fu, Tongming Yin, Jinfeng Cai, Bao Liu, Guihua Lu, Hongyan Lin, Hongwei Han, Zhongling Wen, Jinliang Qi, Yonghua Yang

**Affiliations:** 1 State Key Laboratory of Pharmaceutical Biotechnology, Institute for Plant Molecular Biology, School of Life Sciences, Nanjing University, Nanjing 210023, China; 2 Co-Innovation Center for Sustainable Forestry in Southern China, Nanjing Forestry University, Nanjing 210037, China; 3 Key Laboratory of Molecular Epigenetics of the Ministry of Education (MOE), Northeast Normal University, Changchun 130024, China; 4 School of Life Sciences, Huaiyin Normal University, Huaian 223300, China

**Keywords:** duplication and loss, evolutionary, enzymatic activities, HMGR, *Lithospermum erythrorhizon*

## Abstract

3-hydroxy-3-methylglutaryl-CoA reductase (HMGR), as the rate-limiting enzyme in the mevalonate pathway, is essential for the biosynthesis of shikonin in *Lithospermum erythrorhizon*. However, in the absence of sufficient data, the principles of a genome-wide in-depth evolutionary exploration of *HMGR* family members in plants, as well as key members related to shikonin biosynthesis, remain unidentified. In this study, 124 *HMGR*s were identified and characterized from 36 representative plants, including *L. erythrorhizon*. Vascular plants were found to have more HMGR family genes than nonvascular plants. The phylogenetic tree revealed that during lineage and species diversification, the *HMGR*s evolved independently and intronless *LerHMGR*s emerged from multi-intron *HMGR* in land plants. Among them, *Pinus tabuliformis* and *L. erythrorhizon* had the most *HMGR* gene duplications, with 11 *LerHMGRs* most likely expanded through WGD/segmental and tandem duplications. In seedling roots and M9 cultured cells/hairy roots, where shikonin biosynthesis occurs, *LerHMGR1* and *LerHMGR2* were expressed significantly more than other genes. The enzymatic activities of LerHMGR1 and LerHMGR2 further supported their roles in catalyzing the conversion of HMG-CoA to mevalonate. Our findings provide insight into the molecular evolutionary properties and function of the *HMGR* family in plants and a basis for the genetic improvement of efficiently produced secondary metabolites in *L. erythrorhizon*.

## 1. Introduction

Plant-derived medicinal natural products are important sources of medicines for treating a wide range of diseases. *Lithospermum erythrorhizon* Sieb. et Zucc, an important medicinal plant in East Asian and Western traditional medicine, can biosynthesize red naphthoquinone compounds, namely shikonin and its derivatives, in its root periderm [1]. These compounds have been shown to have anti-HIV [2], antioxidant [3], anti-inflammatory [4,5], wound healing [6], and other pharmacological activities [7,8,9], as well as to trigger cancer cell apoptosis, making them yet another promising natural anti-tumor drug [10,11]. Furthermore, these metabolites are widely used natural raw materials for making cosmetics and dyes that have a high market value.

However, due to the difficulties of cultivating *L. erythrorhizon* and the low amount of root periderm, plant-based production is seriously unable to meet the global demand. Despite the development of the chemical synthesis of shikonin, the yield is only 0.7%, involving 12 reaction steps, and the cost is relatively expensive, making it insufficient for industrial production [12,13,14]. Therefore, it is crucial to increase the production of shikonin and its derivatives by identifying key genes in the biosynthesis pathway, using strategies such as high-yield transgenic cell lines and synthetic biology, and utilizing the “two-stage cultivation system” to produce shikonin and its derivatives for *L. erythrorhizon* cells [15,16] and hairy roots [16,17].

The biosynthetic pathway of shikonin and its derivatives has primarily been elucidated: geranyl pyrophosphate is synthesized via the mevalonate (MVA)/methylerythritol phosphate metabolic pathway [18] and *p*-hydroxybenzoic acid is synthesized via the phenylpropanoid pathway [19] to form 3-geranyl-4-hydroxybenzolic acid under the catalysis of *p*-hydroxybenzoate geranyltransferase (PGT) [20,21,22]. The 3-geranyl-4-hydroxybenzolic acid is then catalyzed by geranylhydroquinone 3”-hydroxylase [23,24], CYP76B101 [25], DSH1/DSH2 [26], LeSAT1 [27], LeAAT1 [27] and some unidentified enzymes to generate the final shikonin and its derivatives via a series of reactions including hydroxylation, oxidation, cyclization, acylation, etc. Since Gaisser et al. [28] and Singh et al. [29] discovered that mevinolin, a specific inhibitor of 3-hydroxy-3-methyl-glutaryl CoA reductase (HMGR), inhibits 98% and 92.82% of the accumulation of shikonin and its derivatives in *L. erythrorhizon* and *Arnebia euchroma* suspension cells, respectively, and that the down-regulation of the *L. erythrorhizon* geranyl diphosphate synthase (*LeGPPS*) gene also results in reduced shikonin production and a decreased expression of mevalonic acid and phenylpropanoid pathway genes [30,31], it has been widely believed that the MVA pathway serves as the primary source of the geranyl pyrophosphate required for the biosynthesis of shikonin and its derivatives. Additionally, as the most critical and first rate-limiting enzyme in the MVA pathway, HMGR can catalyze the conversion of one molecule of 3-hydroxy-3-methylglutary-CoA (HMG-CoA) and two molecules of triphosphopyridine nucleotide (NADPH) into mevalonate [32], which may have a significant and direct effect on the biosynthesis of shikonin and its derivatives. 

Polyploidy, or whole genome duplication (WGD), is a common feature of plant genomes, providing opportunities for the expansion and diversification of all gene families [33]. In the evolutionary process of many plants, *HMGR* gradually became a multi-member gene family, and the expression of its members in different tissues is strongly tied to the accumulation of metabolites [32]. Since *HMGR* was first identified in *Arabidopsis* in 1989 [34], over 600 *HMGR* genes have been identified in over 80 plants [35]. However, only a 433 bp sequence of *HMGR* has been cloned in *L. erythrorhizon* [36], and an in-depth analysis of *HMGR* family members of *L. erythrorhizon* on the genome-wide level, as well as the gene‘s evolutionary process in the *plant kingdom*, has yet to be well conducted. Particularly, the putative effects on shikonin accumulation via differential regulation of the *LerHMGR* genes, as well as which *LerHMGR*(*s*) may be predominantly responsible for the accumulation of shikonin and its derivatives, have yet to be explored. 

In this study, we carried out more comprehensive comparative analyses of the *HMGR* gene family in 36 representative plants, including 11 *LerHMGR* family members, to explore their evolutionary mechanism, and predicted that *LerHMGR1* and *LerHMGR2* may be the important members responsible for the formation of shikonin and its derivatives through their expression pattern, subcellular localization and enzymatic activity assays. This research is expected to provide useful information for understanding the *LerHMGR*s in shikonin biosynthesis in *L. erythrorhizon*, and explore the evolutionary principles of *HMGR* family members in plants at the genome-wide level.

## 2. Results

### 2.1. Identification of the HMGR Gene Family in L. erythrorhizon and 35 Plants 

**Figure 1:**
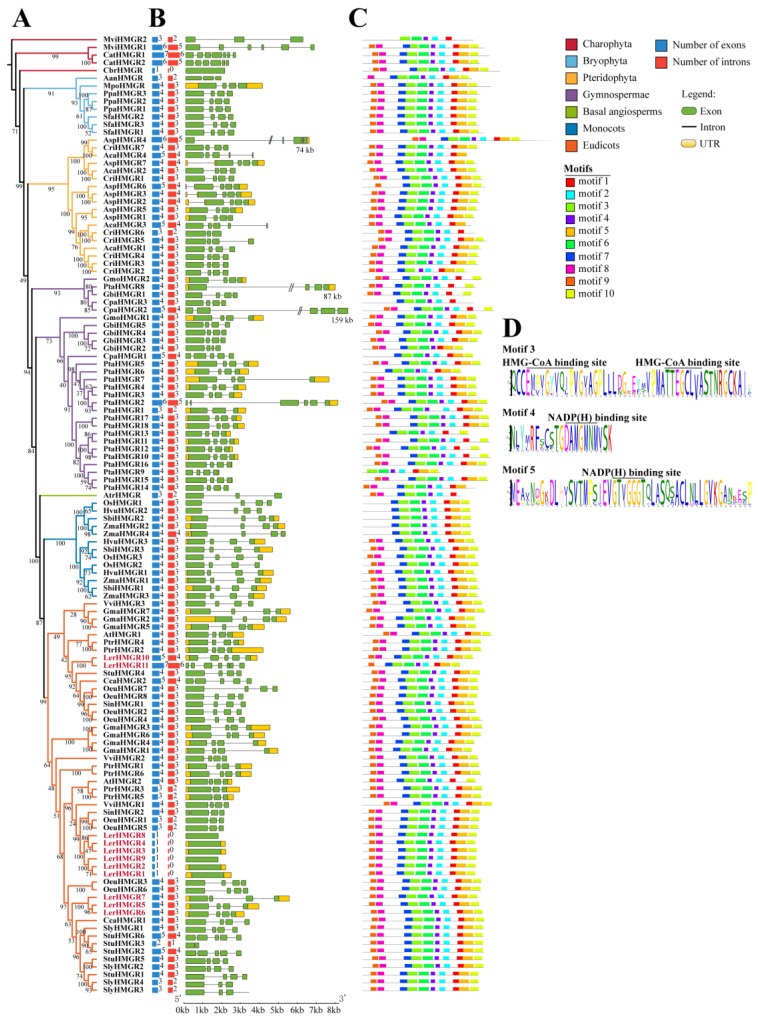
Identification, phylogeny, gene structure, and conserved motif analysis of 124 *HMGR* family genes from 29 representative plants. (**A**) The identification and phylogenetic tree of 124 HMGRs. The phylogenetic tree was constructed based on full-length protein sequences using the maximum likelihood method with 1000 replicates. Mvi: *Mesostigma viride*, Cat: *Chlorokybus atmophyticus*, Cbr: *Chara braunii*, Aan: *Anthoceros angustus*, Mpo: *Marchantia polymorpha*, Ppa: *physcomitrella patens*, Sfa: *Sphagnum fallax*, Asp: *Alsophila spinulosa*, Cri: *Ceratopteris richardii*, Aca: *Adiantum capillus*, Gmo: *Gnetum montanum*, Pta: *Pinus tabuliformis*, Gbi: *Ginkgo biloba*, Cpa: *Cycas panzhihuaensis*, Atr: *Amborella trichopoda*, Osa: *Oryza sativa*, Hvu: *Hordeum vulgare*, Sbi: *Sorghum bicolor*, Zma: *Zea mays*, Vvi: *Vitis vinifera*, Gma: *Glycine max*, Ath: *Arabidopsis thaliana*, Ptr: *Populus trichocarpa*, Ler: *Lithospermum erythrorhizon*, Stu: *Solanum tuberosum*, Cca: *Coffea canephora*, Oeu: *Olea europaea*, Sin: *Sesamum indicum*, Sly: *Solanum lycopersicum*. Pink solid circle represents *LerHMGRs*. (**B**) The number of exons and introns and the gene structure of 124 *HMGRs*. (**C**) The distribution of the conserved motif. The ten conserved motifs were identified using the MEME program. (**D**) Three conserved motif logos including HMG-CoA binding sites and NADP(H) binding sites.

To better understand the evolutionary history and function of *LerHMGRs* in the upstream pathway of shikonin biosynthesis, we identified a total of 124 *HMGR* family genes from the reference genomes of 36 species, including *L. erythrorhizon*, using the previously published HMM model (PF00368) (Figure 1A). All of these species could well represent the main taxa of plants and contained 10 lower plants, 4 Bryophytes, 3 Pteridophyta, 4 Gymnosperms, and 15 Angiosperms (1 basal angiosperm, 10 eudicots, and 4 monocots). More *HMGR* family genes were found in vascular plants than in nonvascular plants. In the genomes of 10 species of Phycophyta, no *HMGR* gene was identified in the genomes of seven species of Chlorophyta, and 1–2 *HMGR* genes were identified in the genomes of three species of Charophyta (Table S1). The *Pinus tabuliformis* was the most *HMGR*-gene-member-rich species among the plants in our analysis, which was expected as it has one of the largest plant genomes known to have ever been assembled [37]. *HMGR* gene numbers vary widely among flowering plants, with five of the ten eudicots having a higher number than monocots (Table S1). The average number of *HMGR* family genes among all species (except Chlorophyta) was 4.28, and 68.96% of species had *HMGR* genes below the average level (Table S1). *L. erythrorhizon* had 11 *HMGRs* and the highest percentage of *HMGR* number genes (Table S1).

### 2.2. Evolution and Characterization of the HMGR Gene Family in Plants

To explore the evolution of the *HMGR* gene family in plants, we constructed a phylogenetic tree using all 124 HMGR protein sequences from 29 plants. The results showed that the taxonomic position of HMGRs in the seven lineages was consistent with the plant’s evolutionary order, indicating that plant HMGRs developed into different branches after the lineages diverged (Figure 1A). LerHMGRs roughly divided into three portions in eudicots lineages, all of which closely related to the Lamiids plant’s HMGRs. LerHMGR5~LerHMGR7 was closely related to 10 members (SlyHMGR3/4/2/1, StuHMGR1/5/2/3/6, CcaHMGR1) from *S. lycopersicum*, *S. tuberosum*, and *C. canephora*; the six LerHMGRs (LerHMGR1~LerHMGR4, LerHMGR8, LerHMGR9) were further classified into two broad categories that are more closely related to Lamiales SinHMGR2, OueHMGR5, and OueHMGR1; the closest member of LerHMGR10 and LerHMGR11 were seven members (OeuHMGR4/2/8/7, SinHMGR1, CcaHMGR2, StuHMGR4). 

Introns are closely related to gene expression, transcription shearing, and other processes, and they may play a key role in plant adaptability and evolution. Gene structure analysis using the GSDS tool predicted that the number of introns in all *HMGRs* ranged from 0 to 6, with 69.35% having three introns (Figure 1B, Table S2). *LerHMGR1-LerHMGR4*, *LerHMGR8*, *LerHMGR9*, and *CbrHMGR* are intronless genes (Figure 1B), implying that intronless *CbrHMGR* has undergone a unique evolution process and that intronless *LerHMGRs* emerged from multi-intron *HMGR* in land plants, and these six *LerHMGR* genes may be more involved in the quick response to stress than other members of the gene family [38]. In addition, we identified an intron in the 5′ UTR region of *AspHMGR2*, *AspHMGR3*, *AspHMGR7*, and *ZmaHMGR4*. These 5′ UTR introns may increase the promoter activity or translation efficiency of the corresponding genes [39,40] (Figure 1B, Table S2). 

Physicochemical properties and the identification of 10 motifs in 124 plant HMGRs showed that most HMGRs were conserved throughout the evolution of lower plants into higher plants (Figure 1C and Figure S1, Table S2). In the catalytic domains, most of them have three conserved motifs: motif 3 represents the two HMG-CoA binding sites (EMPVGYVQIP and TTEGCLVA) and motif 4 and motif 5 represent the two NADP(H) binding sites (DAMGMNM and GTVGGGT) (Figure 1D). Among them, the second NADP(H) binding site was detected in all 12 HMGRs with motif 5 deletion, which may be due to the low conserved flanking sequence of this binding site (Figure 1C). In addition, motif 8 or motif 9, which contain the transmembrane helix typically possessed by plant HMGRs, were not found in the N-terminal region of 15 HMGRs, including one branch of monocots (Figure 1C and Figure S1). Among them, the absence of motif 8 and motif 9 does not correspond to TMHMM Server v.2.0′s prediction due to the presence of one transmembrane helix in MviHMGR2, PtaHMGR6, PtaHMGR13, and SbiHMGR2. This may be due to the diversity of the sequences.

### 2.3. Gene Expansion of HMGR Gene Family in L. erythrorhizon

**Figure 2:**
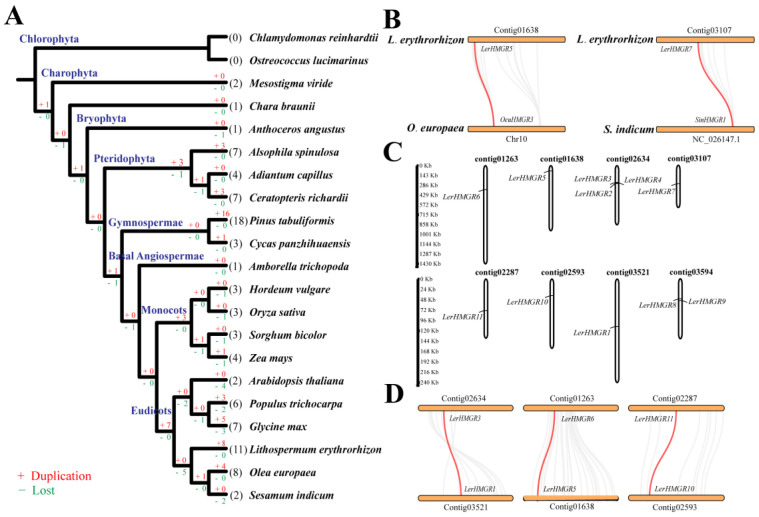
Gene expansion, synteny analysis and genome location of *HMGRs* in *L. erythrorhizon*. (**A**) Gene duplication and loss analysis of *HMGR* family genes using Notung software in 19 representative plants. Gene duplications and losses are represented by the + and − numbers on each branch. The numbers in parentheses in (**A**) represent the number of *HMGR* gene family members identified from different species. (**B**) Synteny blocks of *HMGRs* between *L. erythrorhizon* and *O. europaea*, *S. indicum.* (**C**) Location of *LerHMGRs* in the genome of *L. erythrorhizon.* (**D**) Synteny blocks of six *LerHMGRs*. The red line represents the homologous *HMGRs* between *L. erythrorhizon* and *O. europaea*, *S. indicum*, and *L. erythrorhizon*.

Gene duplications and loss play a significant factor in plant evolution and plant fitness [41]. To further understand the evolution of the *HMGR* gene family, we performed gene duplication and loss analysis in 19 representative plants from seven lineages using Notung software by reconciling gene phylogenetic trees and species taxonomy common trees (Figure 2A). Based on the number of variations found in *HMGR* family genes at different evolutionary stages, *HMGR* ancestral genes were duplicated prior the emergence of *Mesostigma viride*, but none were lost (Figure 2A). No genes were duplicated or lost in the lineage of the common ancestor of two Gymnospermae; however, 16 genes were duplicated in *Pinus tabuliformis* after its divergence from *Cycas panzhihuaensis* (Figure 2A). Similarly, no genes were lost or duplicated in the lineage of the common ancestor of the four monocots and the six eudicots; but, three and seven genes, respectively, were duplicated after the divergence of the ancestors of the monocots and the eudicots (Figure 2A). Moreover, there was a wide range of *HMGR* genes that were duplicated and lost during the evolution of the six eudicots plants. Among the eudicots plants, the *HMGR* genes of *L. erythrorhizon* experienced the most duplications (Figure 2A).

To elucidate the expansion mechanisms of the *LerHMGR* gene family in *L. erythrorhizon*, we analyzed the genomic location and duplication types of each *LerHMGR*. Then the synthetic blocks were identified in the entire *L. erythrorhizon* genome, as well as between it and the *O. europaea* and *S. indicum* genomes. We also calculated the median synonymous substitution rates (Ks) of the syntenic fragments containing *LerHMGR* genes (Table S3). In the results of the synteny analysis between the genomes of *L. erythrorhizon*, *O. europaea*, and *S. indicum*, *LerHMGR5* was collinear with *OeuHMGR3*, while *LerHMGR7* was collinear with *SinHMGR*1 (Figure 2B). It suggested that most *LerHMGRs* were acquired through species-specific duplication events during plant evolution.

The genomic location showed that these 11 *LerHMGRs* were located on eight different contigs (Figure 2C). Four members (i.e., *LerHMGR2*, *LerHMGR4*, *LerHMGR8*, and *LerHMGR9*) might be derived from tandem duplications, as indicated by their genomic loci: *LerHMGR2*, *LerHMGR3*, and *LerHMGR4* were closely located in the contig02634, while *LerHMGR8* and *LerHMGR9* were closely located in the contig03594 (Figure 2C, Table 1). Six members (i.e., *LerHMGR1*, *LerHMGR3*, *LerHMGR5*, *LerHMGR6*, *LerHMGR10*, and *LerHMGR11*) might be derived from WGD/segmental (Table 1). Contig03521 containing *LerHMGR1*, contig01638 containing *LerHMGR5*, and contig02593 containing *LerHMGR10* exhibit synteny relationships with contig02634 containing *LerHMGR3*, contig01263 containing *LerHMGR6*, and contig02287 containing *LerHMGR11*, respectively (Figure 2D).

**Figure 3:**
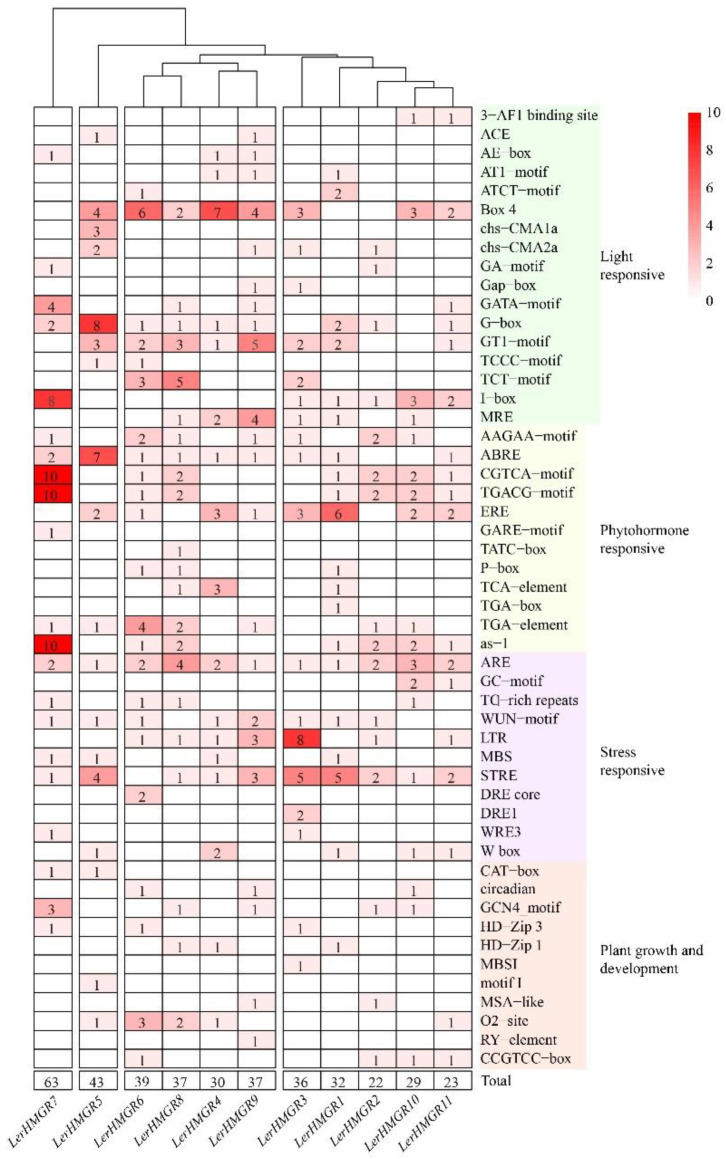
Types and numbers of cis-acting element in the promoters of *LerHMGRs*. Different colors and numbers of the grids indicate the numbers of different types of cis-acting element types in the promoters.

Furthermore, the Ks distribution was analyzed by calculating the Ks value of 8257 synteny gene pairs based on Tang’s published *L. erythrorhizon* genome data [42] (Table S4). It was found that there were two peaks (Ks = about 0.088 and 0.376), indicating that the *L. erythrorhizon* genome may have undergone two rounds of genome-wide duplication (Figure S2). In addition, three syntenic blocks containing *LerHMGR* genes were identified, as shown in Figure 3D. The syntenic block containing *LerHMGR5* and *LerHMGR6* had a median Ks of 0.395, which was approximate to the multi-locus peak (~0.376) in the Ks distribution (Tables S3 and S4). It is possible that the duplications arose via the WGD event. The syntenic block containing *LerHMGR1* and *LerHMGR3* had a median Ks of 0.444, and the syntenic block containing *LerHMGR10* and *LerHMGR11* had a medianKs of 0.053 (Table S3), implying that the *LerHMGR* family may have undergone one ancient and one recent segmental duplication.

### 2.4. Cis-Acting Elements Revealed the Possible Transcription Regulation of LerHMGRs in L. erythrorhizon 

The PlantCARE tool was used to scan the 2000 bp upstream promoter regions of *LerHMGR* genes for putative cis-acting elements that regulate their expression, and a total of 2019 cis-acting elements of 80 types were found in all *LerHMGRs* promoters (Table S5). Among them, 17 types of light-responsive elements were identified, including Box 4, the GA-motif, the G-box, and the MYB binding site involved in light responsiveness (MRE), which are consistent with the photoinhibition regulation of shikonin biosynthesis [43] (Figure 3). Meanwhile, 12 hormone-related cis-elements were identified, with the majority of *LerHMGR* promoters containing responsive binding sites—MeJA-responsive element (CGTCA-motif, TGACG-motif), ethylene-responsive element (ERE), and auxin-responsive element (TGA-element), implying that *LerHMGR* genes may be regulated by jasmonic acid, ethylene, or auxin, all of which have been shown to promote shikonin biosynthesis [44,45,46] (Figure 3). There are 15 distinct types of stress-related elements, including the anaerobic induction regulatory element (ARE), defense- and stress-responsive element (TC-rich repeats), low-temperature responsiveness element (LTR), and the MYB binding site involved in drought-inducibility (MBS), and these stress conditions are analogous to those found in shikonin-producing species (Figure 3). Additionally, the presence of numerous growth-related components suggests that *LerHMGRs* may contribute to the growth of *L. erythrorhizon* (Figure 3). Overall, certain elements such as Box 4, STRE, ARE, TGACG-Box, and ERE were identified as high-frequency elements in the promoters of *LerHMGRs* (Figure S3). Cluster analysis revealed that *LerHMGR2* contained the fewest light-responsive elements and total cis-acting elements (Figure 3). To summarize, these results suggest that transcript levels of *LerHMGRs* may be regulated by components in light, hormone, and stress response regulatory pathways associated with the accumulation of shikonin in *L. erythrorhizon*. 

### 2.5. Expression Patterns Revealed the Possible Critical Role of LerHMGR1 and LerHMGR2 for the Biosynthesis of Shikonin and Its Derivatives

**Figure 4:**
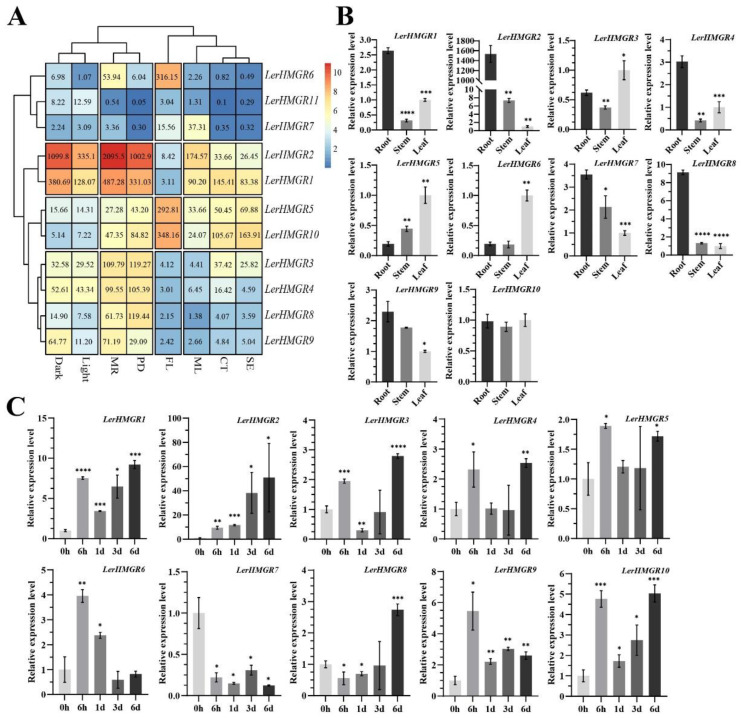
Expression pattern of *LerHMGRs* in different tissues and cultured *L. erythrorhizon* callus cells/hairy roots. (**A**) Heat map showing the expression profile of *LerHMGRs* in different tissues of *L. erythrorhizon* using transcriptome data. Dark: M9 and darkness-cultured hairy roots; Light: B5 and light-cultured hairy roots; MR: mature root; PD: periderm of mature root; FL: flowers; ML: leaves + stems; CT: cortex of mature root; SE: stele of mature root. The number in the grid is the TPM value of each gene in different tissues and under different treatments. The expression values were normalized by Log2 (TPM + 1) to create the heat map, using the average TPM of three biological replicates. (**B**) The expression level of *LerHMGRs* in the leaf, stem, and root of *L. erythrorhizon* was analyzed using qPCR. (**C**) The expression level of *LerHMGRs* in *L. erythrorhizon* cells cultured in M9 and darkness for different times (0 h, 6 h, 1 day, 3 day, and 6 day) was detected using qPCR. The error bars indicate the SDs of three replicates. Asterisks represent significant differences via the Student’s *t*-test analysis (* *p* < 0.05, ** *p* < 0.01, *** *p* < 0.001, **** *p* < 0.0001) as compared with root in (**B**) and 0 h in (**C**), respectively.

Given that the production of secondary metabolites is associated with the tissue-specific expression of related genes [47], and that shikonin accumulates abundantly in the root [48] and M9 in the dark [49], genes involved in the shikonin pathway should be highly expressed under these conditions. We first screened for major *LerHMGRs* involved in shikonin biosynthesis using publicly available transcriptome data from six tissues (the mature root (MR), periderm of mature root (PD), cortex of mature root (CT), stele of mature root (SE), leaves + stems (ML), and flowers (FL)) of the *L. erythrorhizon* seedlings and two growth conditions (wild-type hairy root tissue cultures grown in M9 in the dark versus B5 in the light) [22,50]. The results showed most *LerHMGRs* had expression levels in the mature root, root periderm, and hairy root growing in M9 under darkness, which is the primary site for the accumulation of shikonin and its derivatives, but *LerHMGR2* had the highest level of expression, followed by *LerHMGR1* (Figure 4A). The TPM value of *LerHMGR2* in the mature root and periderm was 19~3880 times and 8.4~20,058 times higher than that of other genes except *LerHMGR1*, respectively (Figure 4A). Additionally, the TPM value of *LerHMGR1* in the mature root was 4.43~902.37 times that of other genes, and that of *LerHMGR1* in periderm was 2.77~6626 times that of other genes (Figure 4A). Furthermore, the TPM values of *LerHMGR2* and *LerHMGR1* in hairy roots under M9 dark culture were 17.18~491.2 times and 5.88~190.34 times those of other genes, respectively (Figure 4A).

Furthermore, due to the inability to obtain the amplification product for *LerHMGR11*, the relative expression levels of the other ten *LerHMGRs* were respectively determined using real-time quantitative PCR (qPCR) in the three tissues (roots, stems, and leaves) of *L. erythrorhizon* seedlings and *L. erythrorhizon* callus cells cultured in M9 in the dark at different time points (0 h, 6 h, 1 day, 3 day, and 6 day). The results roughly corroborated the transcriptome data, where *LerHMGR2* expression was highly induced in the root compared to other homologs (Figure 4B), and its relative expression increased 49.85-fold in the M9 dark culture at 6 day (Figure 4C). *LerHMGR1′*s relative expression increased 8.21-fold in the M9 dark culture (Figure 4C). In contrast, other members only increased by a maximum of 4.45-fold at all testing time points. These findings suggest that *LerHMGR2* and *LerHMGR1* may be the major regulators of shikonin biosynthesis.

### 2.6. LerHMGR1 and LerHMGR2 Are Localized to the Endoplasmic Reticulum 

**Figure 5:**
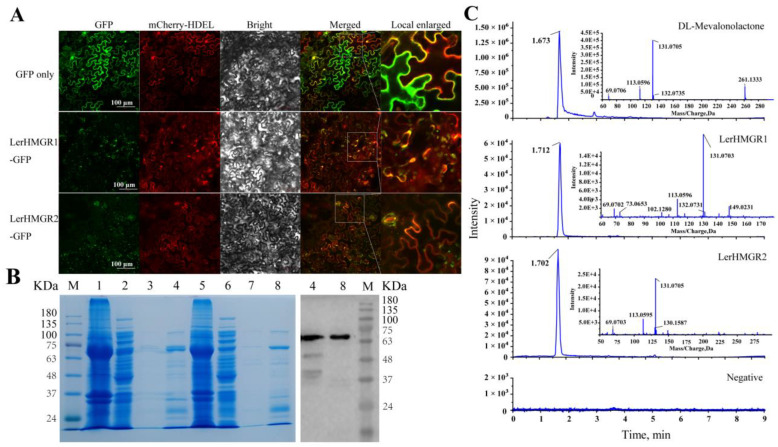
The subcellular localization and catalytic activity of LerHMGR1 and LerHMGR2. (**A**) Subcellular localization of LerHMGR1 and LerHMGR2 in tobacco leaves. The yellow arrow indicates the overlapping location of the proteins. Scale bar = 100 μm. (**B**) SDS-PAGE and Western blot of LerHMGR1 and LerHMGR2 proteins produced through prokaryotic expression. M: protein marker, line 1 and line 5: Cell pellet of LerHMGR1 and LerHMGR2, lines 2 and 6: supernatant of LerHMGR1 and LerHMGR2, lines 3 and 7: His-tag purified-1 LerHMGR1 and LerHMGR2 (Purified-1 means the first tube eluent after Ni-NTA agarose resin purification, and it contains a low concentration of purified protein), lines 4 and 8: His-tag purified-2 LerHMGR1 and LerHMGR2 (Purified-2 means the second tube eluent after Ni-NTA agarose resin purification, and it contains a high concentration of purified protein, which is suitable for follow-up experiments). (**C**) Total ion chromatogram and mass spectra of the enzymatic reaction product. DL-Mevalonolactone is a product formed by the easy esterification of MVA.

Proteins can perform their biological functions only if they have the correct subcellular localization. To confirm whether LerHMGR1 and LerHMGR2 actually have enzymatic activities to catalyze the conversion of HMG-CoA and NADPH into mevalonate, we first transiently expressed LerHMGR1-eGFP and LerHMGR2-eGFP in tobacco leaf cells to determine the subcellular localization. The green fluorescence of the HMGR-eGFP fusion protein was only detected in the endoplasmic reticulum (ER) labeled with the marker protein mCherry-HDEL, whereas the eGFP protein had no specific localization distribution in tobacco cells, which is consistent with the results demonstrating the insertion of *Arabidopsis* HMGR localization into the ER [51]. This suggests that LerHMGR1 and LerHMGR2 are predominantly localized at the ER in plant cell (Figure 5A, Table S2).

### 2.7. Functional Identification of LerHMGR1 and LerHMGR2 In Vitro 

Subsequently, in order to investigate whether LerHMGR1 and LerHMGR2 are active enzymes, LerHMGR1 and LerHMGR2 were expressed heterologously in the *Escherichia coli* strain BL21 (DE3). SDS-PAGE and Western blot can both detect two specific fusion proteins with a molecular weight of approximately 70 KDa (LerHMGR1/LerHMGR2: ~60.4 KDa, two his-tag: ~1.68 KDa, S-tag: ~8.1 KDa) (Figure 5B), indicating that the protein was successfully expressed and isolated from the supernatant of the resultant recombinant *E. coli*. Furthermore, using purified protein, the enzyme abilities of these two LerHMGRs were tested in vitro. The reaction product was analyzed using UPLC-Triple-TOF-MS/MS, and the special ion current peak or mass fragmentation pattern of mevalonolactone (a mevalonate esterification product) was detected at 1.712 and 1.702 min, and the UPLC/Triple TOF 4600+ *m*/*z* values were 131.0703 Da and 131.0705 Da, respectively, which are consistent with the results for mevalonolactone as standard (Figure 5C and Figure S4). However, no distinct peak of mevalonolactone was observed in the controls, and the UPLC/Triple TOF 4600+ *m*/*z* value was different from 131.07 Da (Figure 5C). The results indicated that both LerHMGR1 and LerHMGR2 had the ability to catalyze the conversion of HMG-CoA and NADPH into MVA.

## 3. Discussion

Shikonin extracted from *L. erythrorhizon* has a wide spectrum of medical and commercial values, and its biosynthesis is jointly regulated by various enzymes that are members of multiple gene families, especially HMGR in the upstream MVA pathway, and PGT in the downstream pathway to link the upstream MVA and phenylpropanoid pathways [22,52]. HMGR, which catalyzes the formation of MVA from HMG-CoA, is the first key rate-limiting enzyme in the metabolic synthesis of shikonin and terpenoids [29,52]. In this study, we successfully identified 11 *LerHMGRs* from *L. erythrorhizon* and 113 *HMGR* family genes from the whole genome sequence of the other 35 representative plants, and their characteristics, phylogenetic relationships, duplications and losses, and expansion events were systematically studied. It complements *HMGR* gene family analysis in some species recently sequenced [32]. More importantly, through expression patterns analysis and in vitro enzyme activity, we hypothesized that *LerHMGR1* and *LerHMGR2* play key rate-determining roles in shikonin biosynthesis.

Our analysis revealed that more *HMGR* family genes were detected in higher plants than in lower plants, particularly in *P. tabuliformis*, *L. erythrorhizon*, and *O. europaea*, which may be due to the large number of transposons in Gymnosperms [37] and the multiple WGD or WGT events in Angiosperms [53], leading to the duplication of more *HMGR* genes to adapt to the ecological environment. The analysis of *LerHMGR*’s evolutionary status and expansion events demonstrated the importance of WGD, segmental duplication, and tandem duplication in the evolution of the shikonin/alkannin pathway in *L. erythrorhizon*, which is consistent with other studies of specialized metabolisms in plants [54,55,56]. 

We identified a total of 11 *LerHMGR* family members from the *L. erythrorhizon* genome published by Tang et al. [42], which is contradictory to the number of *LerHMGR* family members we identified from the genome published by Auber et al. in 2020 [22]. Blast analysis results for two *HMGR* group sequences showed that the eight *LerHMGRs* identified from the genome by Auber et al. belong to the eleven *LerHMGR* family members identified from the genome by Tang et al. (Table S6). Additionally, ten *LerHMGRs* sequences were further confirmed via amplification using specific primers. The 433 bp sequence fragment of *HMGR* previously cloned from *L. erythrorhizon* [36] is part of *LerHMGR1* identified in this study. However, *LerHMGR11* could not be amplified using specific primers, and its expression levels in *L. erythrorhizon* roots, stems, and leaves, as well as hairy roots in a B5 light culture and M9 dark culture, were not detected when analyzed by qPCR, which may be due to the low expression level of *LerHMGR11*.

HMGR is mostly found in plants as a gene family [55] and its importance may stem from the fact that it can maintain beneficial metabolite production, that their functional redundancy allows plants to survive adversity or stress, or that each member is in charge of a different final compound synthesis [57]. We investigated the expression patterns of *LerHMGRs* in the tissues and their responses to M9 medium in the darkness (Figure 4). With the exception of *LerHMGR2*, which showed a significant increase in hairy roots/callus cultured in M9 in the darkness compared to B5 in the light, as well as a root-specific expression where shikonin was biosynthesized, *LerHMGR1* also showed an up-regulated expression in the M9 dark culture and root, while the expression levels and induction amplitude of other homologous genes were lower or even had no difference in the M9 dark culture. These findings suggest that *LerHMGR2* and *LerHMGR1* may play a major and redundant role in shikonin biosynthesis. Additionally, we noticed that other *LerHMGRs*, such as *LerHMGR3*~*LerHMGR10*, have an obvious level of expression in leaves, stems, or roots. This implies that the *LerHMGRs* members may have distinct roles in plant growth. For example, *LerHMGR6* is mainly highly expressed in flowers, which may be related to the synthesis of volatile terpenoids or pigment lipid-soluble terpenoids in flowers [58]. *LerHMGR7*, on the other hand, is highly expressed in leaves and may be involved in the accumulation of terpenoids. Similar to the expression levels of the *TmHMGR* gene in *Taxus media* which was generally consistent among leaves, roots, and stems, *LerHMGR10* may also be constitutionally expressed and involved in regulating basic physiological metabolism [59].

*LerHMGR1* and *LerHMGR2* are the only members of the *LerHMGR* gene family that are highly specifically expressed in both the root and M9 dark culture, and their catalytic functions of MVA biosynthesis have been demonstrated by enzyme activity experiments in vitro. Regrettably, we did not clarify the contribution ratio of LerHMGR1 and LerHMGR2 to the accumulation of shikonin. This issue needs to be explored further by comparing the kinetic properties of LerHMGR1 and LerHMGR2, as well as developing transgenic hairy roots with specific gene targets using the Crispr/cas9 approach and overexpression techniques. Furthermore, while the induced expression of all *LerHMGRs* except *LerHMGR1* and *LerHMGR2* was very low, the expression of most genes in B5 to M9 followed a time-dependent pattern of first increasing, then decreasing, and then increasing, which was attributed to the stimulating effect during medium conversion. However, the expression of *LerHMGR2* increased continuously throughout time, suggesting that *LerHMGR2* may play a significant role in the biosynthesis of shikonin in response to various environmental and developmental factors.

The molecular mechanism of the post-translational regulation of HMGR in *Arabidopsis* has been extensively studied: the activity of HMGR1S and HMGR1L is negatively regulated by the phosphorylation of two B-type regulatory subunits of Ser/Thr protein phosphatase 2A (PP2A) phosphatase: B″α and B″β [60,61]. However, research on the transcriptional regulation of HMGR in higher plants is currently restricted. At the transcriptional level, the expression of *HMGR* is regulated by proteins that bind to the sterol regulatory elements of the *HMGR* promoter [62]. To further explore the transcription factors that may regulate the *HMGR* transcript level, we first identified 2419 candidate transcription factors from the *L. erythrorhizon* genome (Table S7), and then performed co-expression network analysis using all transcription factors, *LerHMGR1* and *LerHMGR2*, based on transcriptomes data (Table S8). As shown in Figure S5, LE19274.1, LE10602.1, and LE00798.1, belonging to C2H2, and LE28791.1, belonging to AP2/ERF-ERF, had significant positive correlations with *LerHMGR1* and *LerHMGR2* (r > 0.65, *p* < 0.05), suggesting that they may be transcription factors that positively regulate the *HMGRs*. 

In conclusion, we thoroughly investigated the *HMGR* gene family in 36 representative plants. Exploring the evolutionary history of the gene family and identifying the key school genes involved are a necessary basis for improving the shikonin biosynthesis pathway and increasing shikonin production through genetic engineering. 

## 4. Materials and Methods

### 4.1. Plant Materials and Treatment

*Lithospermum erythrorhizon* Sieb. et Zucc seeds collected in Inner Mongolia, China, were allowed to germinate on moist sand in the dark at 4 °C for about one month. Plants were grown on soil in growth chambers at 25°C with a 16 h day/8 h night photoperiod, 100 μE·m^−2^·s^−1^ light intensity, and 60–70% relative humidity. The *L. erythrorhizon* suspension culture cell lines were made from the radicle of *L. erythrorhizon.* They were grown in B5 medium in the light and then shikonin production was induced in M9 medium in the darkness. 

### 4.2. Identification of HMGR Family Genes

The Pfam domain PF00368 is found in Pfam protein family databases (http://pfam.xfam.org/, accessed on 25 April 2023) and HMMER 3.0 (https://github.com/PhyreEngine/conda-hmmer, accessed on 25 April 2023) was used to search the genomes of 36 plants for *HMGR* genes. The download addresses for genome files of all species are listed in Table S1. Then redundant sequences and abnormal sequences (incomplete PF00368 domain) identified by Batch CD-Search (https://www.ncbi.nlm.nih.gov/Structure/bwrpsb/bwrpsb.cgi, accessed on 25 April 2023) were removed. Eleven gene sequences of the *HMGR* family in *L. erythrorhizon* genome were obtained, then used to blast against the eight *LerHMGRs* from the genome published by Auber et al. using all-to-all blastp, and primers (Table S9) were designed for amplification with cDNA mixtures of seedling leaves and roots as template to verify all sequences in *L. erythrorhizon* using Phanta Max Master Mix (Vazyme, #P515, Nanjing, China). 

### 4.3. Bioinformatics Analysis

For HMGR phylogenetic tree construction, the amino acid sequences of the identified *HMGR* family members were aligned via MAFFT v7.310 [63]. Subsequently, the preliminary alignment was trimmed using trimAL v.1.2.rev59 (key parameter: −gt 0.50) [64]. The trimmed alignment was used to construct the phylogenetic tree using IQ-TREE multicore version 2.0.3 and 1000 bootstrap replications [65]. The conserved motif analysis was performed in the MEME program (https://meme-suite.org/meme/tools/meme, accessed on 25 April 2023) using full-length amino acid sequences, as the default setting was 10 for the motif number. The number of introns and exons was analyzed using the Gene Structure Display Server 2.0 (http://gsds.gao-lab.org/, accessed on 25 April 2023). The cis-acting elements of the promoter region (2000 bp sequence upstream of the gene) of the *LerHMGR* genes were analyzed using the PlantCARE program (http://bioinformatics.psb.ugent.be/webtools/plantcare/html/, accessed on 25 April 2023). The physicochemical properties, including theoretical Mw, theoretical pI, aliphatic index, and grand average of hydropathicity of HMGRs, were analyzed by Ex-PaSy (http://web.expasy.org/protparam/, accessed on 25 April 2023). The prediction of transmembrane helices in HMGR was performed using the TMHMM Server v.2.0 (https://services.healthtech.dtu.dk/services/TMHMM-2.0/, accessed on 25 April 2023). Gene synteny and duplication types of *HMGRs* were analyzed using the ‘MCScanX’ and ‘duplicate_gene_classifier’ programs implemented in the MCScanX package [66]. The Ks of gene pairs were calculated with TBtools software (V1.098696) using the Simple Ka/Ks calculator [67]. For the Ks frequency distribution, MCScanX [66] was used to identify synthetic blocks within the genome of *L. erythrorhizon* and extract information on synteny gene pairs. Then, the wgd package (https://github.com/arzwa/wgd, accessed on 25 April 2023) was used to calculate the Ks values between synteny gene pairs, and ggplot2 (https://github.com/tidyverse/ggplot2, accessed on 25 April 2023) was used to plot histograms and fit curves (Ks values less than 0.05 and greater than 5 were filtered out).

### 4.4. Duplication/Loss Detection of HMGR Gene Family

The phylogenetic trees of 21 species and their HMGRs were input into the Notung-2.9.1.5 software [68] for the detection of duplications and loss of *HMGR* family genes, respectively. The phylogenetic trees of 21 species were made using the iTOL (https://itol.embl.de/, accessed on 25 April 2023) according to the relationship of species in NCBI taxonomy [69].

### 4.5. RNA-Seq Experiments

RNA-seq data from *L. erythrorhizon* whole root (MR), root periderm (PD), root cortex (CT), root stele (SE), leaves + stems (ML), flowers (FL), and hairy roots grown in M9 in the dark and B5 in the light were downloaded and used for gene expression analysis from the NCBI SRA (accession ID: SRP141330, SAMN13650849, SAMN13650867) [22,50]. The expression level of each gene was calculated using RNA sequencing quantification analysis with the transcripts per kilobase of the exon model per million mapped reads method by TPMCalculator [70]. A log2(TPM + 1) value was used to quantify the expression level of each *LerHMGR* gene, and a clustering heatmap was drawn in R (package: pheatmap).

### 4.6. RNA Extraction and RT-qPCR Analysis

Total RNA was extracted from cultured cells, hairy roots, and different tissues of *L. erythrorhizon* using the FastPure Plant Total RNA Isolation Kit (Vazyme, #RC401, Nanjing, China). The RNA purity and integrity were assessed based on the A260/A280 absorbance ratio and 1.0% agarose gel electrophoresis. cDNA was synthesized by reverse transcription with the HiScript III 1st Strand cDNA Synthesis Kit (+gDNA wiper) (Vazyme, #R312), and qPCR was performed using the ChamQ Universal SYBR qPCR Master Mix (Vazyme, #Q711) with gene-specific primers (Table S9) on an Applied Biosystems 7500 Real-Time PCR System and StepOnePlus™ Real-Time PCR System. Gene expression levels of each sample were normalized relative to *GAPDH* mRNA as an internal standard and calculated using the 2^−ΔΔCt^ method [71]. At least three independent experiments were performed for each analysis.

### 4.7. Heterologous Expression of LerHMGR1 and LerHMGR2 in Escherichia coli

The coding sequence (CDS) of *LerHMGR1* and *LerHMGR2* were subcloned into the *Bam*H I/*Hin*d III sites of the prokaryotic expression vector pET-30a (+) and transformed into *E. coli BL21*(DE3) using homologous recombination (Vazyme, #C115). After growing the transformants to an OD_600_ of 0.6 in 400 mL of lysogeny broth medium at 37 °C, protein expression was induced by adding 0.5 mM of isopropyl β-D-1-thiogalactopyranoside, followed by 20 h of cultivation at 25 °C. The primers used for plasmid construction are listed in Table S9.

After centrifugation, the bacteria were resuspended in 32 mL of 1 × PBS and 1 mM PMSF was added, and ultrasonic crushing was performed for 30 min. Then the bacteria debris was removed by centrifugation at 10,000 *g* for 20 min. The supernatant was applied to an affinity column filled with 3 mL of Ni-NTA Agarose Resin (Yeasen, #20502ES10, Shanghai, China), and the protein was purified following the manufacturer’s instructions. Purified fusion proteins were eluted in an elution buffer containing 50 mM NaH_2_PO_4_ (pH 8.0), 300 mM NaCl, and 250 mM imidazole. They were then used for 10% SDS–PAGE and Western blotting was performed with anti-his mouse monoclonal antibody as the primary antibody (TransGen Biotech, #HT501, Beijing, China) and goat anti-mouse IgG, HRP conjugate (TransGen Biotech, #HS201) as the secondary antibody.

### 4.8. In Vitro Enzyme Activity Assay

The enzymatic assays for LerHMGRs were carried out in the manner described by Wilding et al. [72], with minor modifications. A total of 10 μg His-tag purified protein was added to 1 mL of assay buffer (25 mM K_2_HPO_4_, 50 mM KCl, 1 mM EDTA, 5 mM DTT, 0.3 mM NADPH, 0.3 mM HMG-CoA, and pH 7.5) and the reaction system was incubated at 30 °C for 30 min. To stop the enzymatic reaction, 100 μL HCl (6 M) was added to each reaction system and the products were lactonized for 1 h at 25 °C. The products were extracted in 2 mL of ethyl acetate, which was then evaporated and resuspended in 200 μL ethanol absolute. The final products were analyzed using a UPLC-Triple-TOF-MS/MS system (AB SCIEX, Framingham, MA, USA) with a Welch Ultimate XB-C18 column and separation conditions. A DL-Mevalolactone was purchased from Shanghai Yuanye Bio-Tec (Shanghai, China) as the standard. Additionally, a negative control with sterilized water in place of the target LerHMGR protein was added to the reaction mixture for identification. 

### 4.9. Subcellular Localization Analysis

The subcellular localization of LerHMGRs was predicted in the endoplasmic reticulum by ProtComp v. 9.0 (http://linux1.softberry.com/berry.phtml?topic=protcomppl&group=programs&subgroup=proloc, accessed on 25 April 2023). Then, using homologous recombination, the CDS sequences of *LerHMGR1* and *LerHMGR2* were subcloned into the *Xba* I/*Bam*H I sites of vector pBI121::eGFP to generate pBI121::*LerHMGR1*-eGFP and pBI121::*LerHMGR2*-eGFP. A plasmid expressing the ER-mCherry-HDEL protein was used as an endoplasmic reticulum localization marker. At 1:1, *A. tumefaciens* strain GV3101 containing the fusion expression vector (OD_600_ = 1.5) and the same strain containing the ER-mCherry-HDEL vector (OD_600_ = 1.5) were simultaneously injected into the leaves of 4-week-old *Nicotiana tabacum* plants. The fluorescence signal was observed after 72 h using an LSM 880 (Zeiss, Oberkohen, Germany) confocal microscope equipped with an AxioObserver. LerHMGRs-GFP fusion proteins and free GFP were observed in the excitation wavelengths of 488 nm, and mCherry-HDEL was observed in the excitation wavelengths of 561 nm as an ER marker using a Zeiss LSM880 (confocal laser scanning microscope, CLSM). The vector pBI121:: eGFP was stored in our laboratory and the ER-mCherry-HDEL vector was purchased from the MiaoLing Plasmid Platform (http://www.miaolingbio.com/, accessed on 25 April 2023). The primers used for plasmid construction are listed in Table S9.

### 4.10. Co-Expression Network of Transcription Factors and LerHMGR1 and LerHMGR2 Genes 

Transcription factors in *L. erythrorhizon* were identified by using the ITAK (http://itak.feilab.net/cgi-bin/itak/index.cgi, accessed on 25 April 2023). The expression patterns of 69 types of transcription factors, as well as *LerHMGR1* and *LerHMGR2*, were used for performing the co-expression analysis in the R software using the corr.test () function with Pearson’s method. The co-expression network was visualized using Cytoscape_v3.9.1 [73].

## Figures and Tables

**Table 1:** Duplication type identification results of *HMGRs* in *L. erythrorhizon*.

Gene Name	Duplication Types
*LerHMGR1*	WGD/Segmental duplication
*LerHMGR2*	Tandem duplication
*LerHMGR3*	WGD/Segmental duplication
*LerHMGR4*	Tandem duplication
*LerHMGR5*	WGD/Segmental duplication
*LerHMGR6*	WGD/Segmental duplication
*LerHMGR7*	Dispersed duplication
*LerHMGR8*	Tandem duplication
*LerHMGR9*	Tandem duplication
*LerHMGR10*	WGD/Segmental duplication
*LerHMGR11*	WGD/Segmental duplication

## Data Availability

All relevant data are presented within the paper and its Supplementary Files.

## Supplementary Materials

The following supporting information can be downloaded at: https://www.mdpi.com/article/10.3390/ijms241512532/s1.

## References

[1] Tang W., Eisenbrand G. (1992). *Lithospermum erythrorhizon* Sieb. et Zucc. Chinese Drugs of Plant Origin.

[2] Chen X., Yang L., Zhang N., Turpin J.A., Buckheit R.W., Osterling C., Oppenheim J.J., Howard O.M.Z. (2003). Shikonin, a Component of Chinese Herbal Medicine, Inhibits Chemokine Receptor Function and Suppresses Human Immunodeficiency Virus Type 1. Antimicrob. Agents Chemother..

[3] Skrzypczak A., Przystupa N., Zgadzaj A., Parzonko A., Sykłowska-Baranek K., Paradowska K., Nałęcz-Jawecki G. (2015). Antigenotoxic, anti-photogenotoxic and antioxidant activities of natural naphthoquinone shikonin and acetylshikonin and Arnebia euchroma callus extracts evaluated by the umu-test and EPR method. Toxicol. Vitr..

[4] Fan C., Zhang X., Upton Z. (2018). Anti-inflammatory effects of shikonin in human periodontal ligament cells. Pharm. Biol..

[5] Liang D., Sun Y., Shen Y., Li F., Song X., Zhou E., Zhao F., Liu Z., Fu Y., Guo M. (2013). Shikonin exerts anti-inflammatory effects in a murine model of lipopolysaccharide-induced acute lung injury by inhibiting the nuclear factor-kappaB signaling pathway. Int. Immunopharmacol..

[6] Papageorgiou V.P., Assimopoulou A., Ballis A.C. (2008). Alkannins and Shikonins: A New Class of Wound Healing Agents. Curr. Med. Chem..

[7] Yang Y., Wang J., Yang Q., Wu S., Yang Z., Zhu H., Zheng M., Liu W., Wu W., He J. (2014). Shikonin inhibits the lipopolysaccharide-induced release of HMGB1 in RAW264.7 cells via IFN and NF-κB signaling pathways. Int. Immunopharmacol..

[8] Balkwill F., Mantovani A. (2001). Inflammation and cancer: Back to Virchow?. Lancet.

[9] Sun Q., Gong T., Liu M., Ren S., Yang H., Zeng S., Zhao H., Chen L., Ming T., Meng X. (2021). Shikonin, a naphthalene ingredient: Therapeutic actions, pharmacokinetics, toxicology, clinical trials and pharmaceutical researches. Phytomedicine.

[10] Gernapudi R., Duru N., Zhou Q. (2014). Chemopreventive Activities of Shikonin in Breast Cancer. Biochem. Pharmacol. Open Access.

[11] Zhu J., Zhao L., Luo B., Sheng W. (2019). Shikonin regulates invasion and autophagy of cultured colon cancer cells by inhibiting yes-associated protein. Oncol. Lett..

[12] Malik S., Bhushan S., Sharma M., Ahuja P.S. (2014). Biotechnological approaches to the production of shikonins: A critical review with recent updates. Crit. Rev. Biotechnol..

[13] Yazaki K. (2017). Lithospermum erythrorhizon cell cultures: Present and future aspects. Plant Biotechnol..

[14] Terada A., Tanoue Y., Hatada A., Sakamoto H. (1983). Total synthesis of shikalkin [(±)-shikonin]. J. Chem. Soc. Chem. Commun..

[15] Fujita Y., Hara Y., Suga C., Morimoto T. (1981). Production of shikonin derivatives by cell suspension cultures of Lithospermum erythrorhizon. Plant Cell Rep..

[16] Fujita Y., Tabata M., Nishi A., Yamada Y. New medium and production of secondary compounds with the two-staged cul-ture method. Proceedings of the Plant tissue culture 1982: Proceedings, 5th International Congress of Plant Tissue and Cell.

[17] Shimomura K., Sudo H., Saga H., Kamada H. (1991). Shikonin production and secretion by hairy root cultures of Lithospermum erythrorhizon. Plant Cell Rep..

[18] Lichtenthaler H.K., Rohmer M., Schwender J. (1997). Two independent biochemical pathways for isopentenyl diphosphate and isoprenoid biosynthesis in higher plants. Physiol. Plant..

[19] Inouye H., Ueda S., Inoue K., Matsumura H. (1979). Biosynthesis of shikonin in callus cultures of Lithospermum erythrorhizon. Phytochemistry.

[20] Heide L., Tabata M. (1987). Geranylpyrophosphate: P-hydroxybenzoate geranyltransferase activity in extracts of Lithospermum erythrorhizon cell cultures. Phytochemistry.

[21] Yazaki K., Kunihisa M., Fujisaki T., Sato F. (2002). Geranyl Diphosphate:4-Hydroxybenzoate Geranyltransferase fromLithospermum erythrorhizon. J. Biol. Chem..

[22] Auber R.P., Suttiyut T., McCoy R.M., Ghaste M., Crook J.W., Pendleton A.L., Widhalm J.R., Wisecaver J.H. (2020). Hybrid de novo genome assembly of red gromwell (Lithospermum erythrorhizon) reveals evolutionary insight into shikonin biosynthesis. Hortic. Res..

[23] Yamamoto H., Inoue K., Li S.-M., Heide L. (2000). Geranylhydroquinone 3″-hydroxylase, a cytochrome P-450 monooxygenase from Lithospermum erythrorhizon cell suspension cultures. Planta.

[24] Wang S., Wang R., Liu T., Lv C., Liang J., Kang C., Zhou L., Guo J., Cui G., Zhang Y. (2018). CYP76B74 Catalyzes the 3″-Hydroxylation of Geranylhydroquinone in Shikonin Biosynthesis. Plant Physiol..

[25] Song W., Zhuang Y., Liu T. (2020). Potential role of two cytochrome P450s obtained from Lithospermum erythrorhizon in catalyzing the oxidation of geranylhydroquinone during Shikonin biosynthesis. Phytochemistry.

[26] Song W., Zhuang Y., Liu T. (2021). CYP82AR Subfamily Proteins Catalyze C-1′ Hydroxylations of Deoxyshikonin in the Biosynthesis of Shikonin and Alkannin. Org. Lett..

[27] Oshikiri H., Watanabe B., Yamamoto H., Yazaki K., Takanashi K. (2020). Two BAHD Acyltransferases Catalyze the Last Step in the Shikonin/Alkannin Biosynthetic Pathway. Plant Physiol..

[28] Gaisser S., Heide L. (1996). Inhibition and regulation of shikonin biosynthesis in suspension cultures of Lithospermum. Phytochemistry.

[29] Singh R.S., Gara R.K., Bhardwaj P.K., Kaachra A., Malik S., Kumar R., Sharma M., Ahuja P.S., Kumar S. (2010). Expression of 3-hydroxy-3-methylglutaryl-CoA reductase, p-hydroxybenzoate-m-geranyltransferase and genes of phenylpropanoid pathway exhibits positive correlation with shikonins content in arnebia [Arnebia euchroma (Royle) Johnston]. BMC Mol. Biol..

[30] Ueoka H., Sasaki K., Miyawaki T., Ichino T., Tatsumi K., Suzuki S., Yamamoto H., Sakurai N., Suzuki H., Shibata D. (2020). A Cytosol-Localized Geranyl Diphosphate Synthase from *Lithospermum erythrorhizon* and Its Molecular Evolution. Plant Physiol..

[31] Suttiyut T., Auber R.P., Ghaste M., Kane C.N., McAdam S.A.M., Wisecaver J.H., Widhalm J.R. (2022). Integrative analysis of the shikonin metabolic network identifies new gene connections and reveals evolutionary insight into shikonin biosynthesis. Hortic. Res..

[32] Li W., Wei H., He Q., Chen J., Zhang B., Zhu S. (2014). Species-Specific Expansion and Molecular Evolution of the 3-hydroxy-3-methylglutaryl Coenzyme A Reductase (HMGR) Gene Family in Plants. PLoS ONE.

[33] Soltis P.S., Soltis D.E. (2016). Ancient WGD events as drivers of key innovations in angiosperms. Curr. Opin. Plant Biol..

[34] Caelles C., Ferrer A., Hegardt F.G., Boronat A., Balcells L. (1989). Isolation and structural characterization of a cDNA encoding Arabidopsis thaliana 3-hydroxy-3-methylglutaryl coenzyme A reductase. Plant Mol. Biol..

[35] Zheng T., Guan L., Yu K., Haider M.S., Nasim M., Liu Z., Li T., Zhang K., Jiu S., Jia H. (2021). Expressional diversity of grapevine 3-Hydroxy-3-methylglutaryl-CoA reductase (VvHMGR) in different grapes genotypes. BMC Plant Biol..

[36] Lange B.M., Severin K., Bechthold A., Heide L. (1998). Regulatory role of microsomal 3-hydroxy-3-methylglutaryl-coenzyme A reductase for shikonin biosynthesis in Lithospermum erythrorhizon cell suspension cultures. Planta.

[37] Niu S., Li J., Bo W., Yang W., Zuccolo A., Giacomello S., Chen X., Han F., Yang J., Song Y. (2021). The Chinese pine genome and methylome unveil key features of conifer evolution. Cell.

[38] Liu H., Lyu H., Zhu K., Van de Peer Y., Cheng Z. (2021). The emergence and evolution of intron-poor and intronless genes in intron-rich plant gene families. Plant J..

[39] Laxa M., Müller K., Lange N., Doering L., Pruscha J.T., Peterhänsel C. (2016). The 5′UTR Intron of Arabidopsis GGT1 Aminotransferase Enhances Promoter Activity by Recruiting RNA Polymerase II. Plant Physiol..

[40] Kamo K., Kim A.-Y., Park S.H., Joung Y.H. (2012). The 5′UTR-intron of the Gladiolus polyubiquitin promoter GUBQ1 enhances translation efficiency in Gladiolus and Arabidopsis. BMC Plant Biol..

[41] Fischer I., Diévart A., Droc G., Dufayard J.-F., Chantret N. (2016). Evolutionary Dynamics of the Leucine-Rich Repeat Receptor-Like Kinase (LRR-RLK) Subfamily in Angiosperms. Plant Physiol..

[42] Tang C. (2021). Exploring the evolutionary process of alkannin/shikonin *O*-acyltransferases by a reliable *Lithospermum erythrorhizon* genome. DNA Res..

[43] Malik S., Bhushan S., Sharma M., Ahuja P.S. (2011). Physico-chemical factors influencing the shikonin derivatives production in cell suspension cultures of *Arnebia euchroma*(Royle) Johnston, a medicinally important plant species. Cell Biol. Int..

[44] Hao H., Lei C., Dong Q., Shen Y., Chi J., Ye H., Wang H. (2014). Effects of Exogenous Methyl Jasmonate on the Biosynthesis of Shikonin Derivatives in Callus Tissues of Arnebia euchroma. Appl. Biochem. Biotechnol..

[45] Fang R., Wu F., Zou A., Zhu Y., Zhao H., Liao Y., Tang R.-J., Yang T., Pang Y., Wang X. (2016). Transgenic analysis reveals LeACS-1 as a positive regulator of ethylene-induced shikonin biosynthesis in Lithospermum erythrorhizon hairy roots. Plant Mol. Biol..

[46] Bagheri F., Tahvilian R., Karimi N., Chalabi M., Azami M. (2018). Shikonin Production by Callus Culture of Onosma bulbotrichom as Active Pharmaceutical Ingredient. Iran J. Pharm. Res..

[47] Wang X., Hu H., Wu Z., Fan H., Wang G., Chai T., Wang H. (2021). Tissue-specific transcriptome analyses reveal candidate genes for stilbene, flavonoid and anthraquinone biosynthesis in the medicinal plant Polygonum cuspidatum. BMC Genom..

[48] Wu F.-Y., Tang C.-Y., Guo Y.-M., Bian Z.-W., Fu J.-Y., Lu G.-H., Qi J.-L., Pang Y.-J., Yang Y.-H. (2017). Transcriptome analysis explores genes related to shikonin biosynthesis in Lithospermeae plants and provides insights into Boraginales’ evolutionary history. Sci. Rep..

[49] Zhang W.-J., Su J., Tan M.-Y., Liu G.-L., Pang Y.-J., Shen H.-G., Qi J.-L., Yang Y. (2010). Expression analysis of shikonin-biosynthetic genes in response to M9 medium and light in Lithospermum erythrorhizon cell cultures. Plant Cell Tissue Organ Cult. (PCTOC).

[50] Tang C., Li S., Wang Y., Wang X. (2019). Comparative genome/transcriptome analysis probes Boraginales’ phylogenetic position, WGDs in Boraginales, and key enzyme genes in the alkannin/shikonin core pathway. Mol. Ecol. Resour..

[51] Leivar P., González V.M., Castel S., Trelease R.N., López-Iglesias C., Arró M., Boronat A., Campos N., Ferrer A., Fernàndez-Busquets X. (2005). Subcellular Localization of Arabidopsis 3-Hydroxy-3-Methylglutaryl-Coenzyme A Reductase. Plant Physiol..

[52] Song A.A.-L., Abdullah J.O., Abdullah M.P., Shafee N., Othman R., Tan E.-F., Noor N.M., Raha A.R. (2012). Overexpressing 3-Hydroxy-3-Methylglutaryl Coenzyme A Reductase (HMGR) in the Lactococcal Mevalonate Pathway for Heterologous Plant Sesquiterpene Production. PLoS ONE.

[53] Rao G., Zhang J., Liu X., Lin C., Xin H., Xue L., Wang C. (2021). De novo assembly of a new Olea europaea genome accession using nanopore sequencing. Hortic. Res..

[54] Moghe G.D., Leong B.J., Hurney S.M., Jones A.D., Last R.L. (2017). Evolutionary routes to biochemical innovation revealed by integrative analysis of a plant-defense related specialized metabolic pathway. Elife.

[55] Liu W., Zhang Z., Zhu W., Ren Z., Wang Z., Li L., Jia L., Zhu S., Ma Z. (2018). Genome-Wide Identification and Comparative Analysis of the 3-Hydroxy-3-methylglutaryl Coenzyme A Reductase (HMGR) Gene Family in Gossypium. Molecules.

[56] Zhu Y., Wu N., Song W., Yin G., Qin Y., Yan Y., Hu Y. (2014). Soybean (Glycine max) expansin gene superfamily origins: Segmental and tandem duplication events followed by divergent selection among subfamilies. BMC Plant Biol..

[57] Stermer B.A., Bianchini G.M., Korth K.L. (1994). Regulation of HMG-CoA reductase activity in plants. J. Lipid Res..

[58] Li R., Li Z., Leng P., Hu Z., Wu J., Dou D. (2021). Transcriptome sequencing reveals terpene biosynthesis pathway genes accounting for volatile terpene of tree peony. Planta.

[59] Liao Z., Tan Q., Chai Y., Zuo K., Chen M., Gong Y., Wang P., Pi Y., Tan F., Sun X. (2004). Cloning and characterisation of the gene encoding HMG-CoA reductase from Taxus media and its functional identification in yeast. Funct. Plant Biol..

[60] Leivar P., Antolín-Llovera M., Ferrero S., Closa M., Arró M., Ferrer A., Boronat A., Campos N. (2011). Multilevel Control of *Arabidopsis* 3-Hydroxy-3-Methylglutaryl Coenzyme A Reductase by Protein Phosphatase 2A. Plant Cell.

[61] Robertlee J., Kobayashi K., Tang J., Suzuki M., Muranaka T. (2018). Evidence that the Arabidopsis thaliana 3-hydroxy-3-methylglutaryl-CoA reductase 1 is phosphorylated at Ser577 in planta. Plant Biotechnol..

[62] Espenshade P.J., Hughes A.L. (2007). Regulation of Sterol Synthesis in Eukaryotes. Annu. Rev. Genet..

[63] Katoh K., Standley D.M. (2013). MAFFT Multiple Sequence Alignment Software Version 7: Improvements in Performance and Usability. Mol. Biol. Evol..

[64] Capella-Gutiérrez S., Silla-Martínez J.M., Gabaldón T. (2009). trimAl: A tool for automated alignment trimming in large-scale phylogenetic analyses. Bioinformatics.

[65] Minh B.Q., Schmidt H.A., Chernomor O., Schrempf D., Woodhams M.D., von Haeseler A., Lanfear R. (2020). IQ-TREE 2: New Models and Efficient Methods for Phylogenetic Inference in the Genomic Era. Mol. Biol. Evol..

[66] Wang Y., Tang H., DeBarry J.D., Tan X., Li J., Wang X., Lee T.-H., Jin H., Marler B., Guo H. (2012). *MCScanX*: A toolkit for detection and evolutionary analysis of gene synteny and collinearity. Nucleic Acids Res..

[67] Chen C.J., Chen H., Zhang Y., Thomas H.R., Frank M.H., He Y.H., Xia R. (2020). TBtools: An Integrative Toolkit Developed for Interactive Analyses of Big Biological Data. Mol. Plant.

[68] Stolzer M., Lai H., Xu M., Sathaye D., Vernot B., Durand D. (2012). Inferring duplications, losses, transfers and incomplete lineage sorting with nonbinary species trees. Bioinformatics.

[69] Yu T., Bai Y., Liu Z., Wang Z., Yang Q., Wu T., Feng S., Zhang Y., Shen S., Li Q. (2022). Large-scale analyses of heat shock transcription factors and database construction based on whole-genome genes in horticultural and representative plants. Hortic. Res..

[70] Alvarez R.V., Pongor L.S., Mariño-Ramírez L., Landsman D. (2018). TPMCalculator: One-step software to quantify mRNA abundance of genomic features. Bioinformatics.

[71] Livak K.J., Schmittgen T.D. (2001). Analysis of relative gene expression data using real-time quantitative PCR and the 2^−ΔΔCT^ Method. Methods.

[72] Wilding E.I., Kim D.-Y., Bryant A.P., Gwynn M.N., Lunsford R.D., McDevitt D., Myers J.E., Rosenberg M., Sylvester D., Stauffacher C.V. (2000). Essentiality, Expression, and Characterization of the Class II 3-Hydroxy-3-Methylglutaryl Coenzyme A Reductase of *Staphylococcus aureus*. J. Bacteriol..

[73] Shannon P., Markiel A., Ozier O., Baliga N.S., Wang J.T., Ramage D., Amin N., Schwikowski B., Ideker T. (2003). Cytoscape: A software environment for integrated models of Biomolecular Interaction Networks. Genome Res..

