# Supplementary material for: Genome-Wide Comparison and Functional Characterization of HMGR Gene Family Associated with Shikonin Biosynthesis in Lithospermum erythrorhizon
Source: Int J Mol Sci. 2023 Aug 7;24(15):12532. doi: 10.3390/ijms241512532 (PMC10419935; doi:10.3390/ijms241512532)
Supplement: Supplementary file 1 [file ijms-24-12532-s001.zip › Supplementary Figures-R2.pdf]

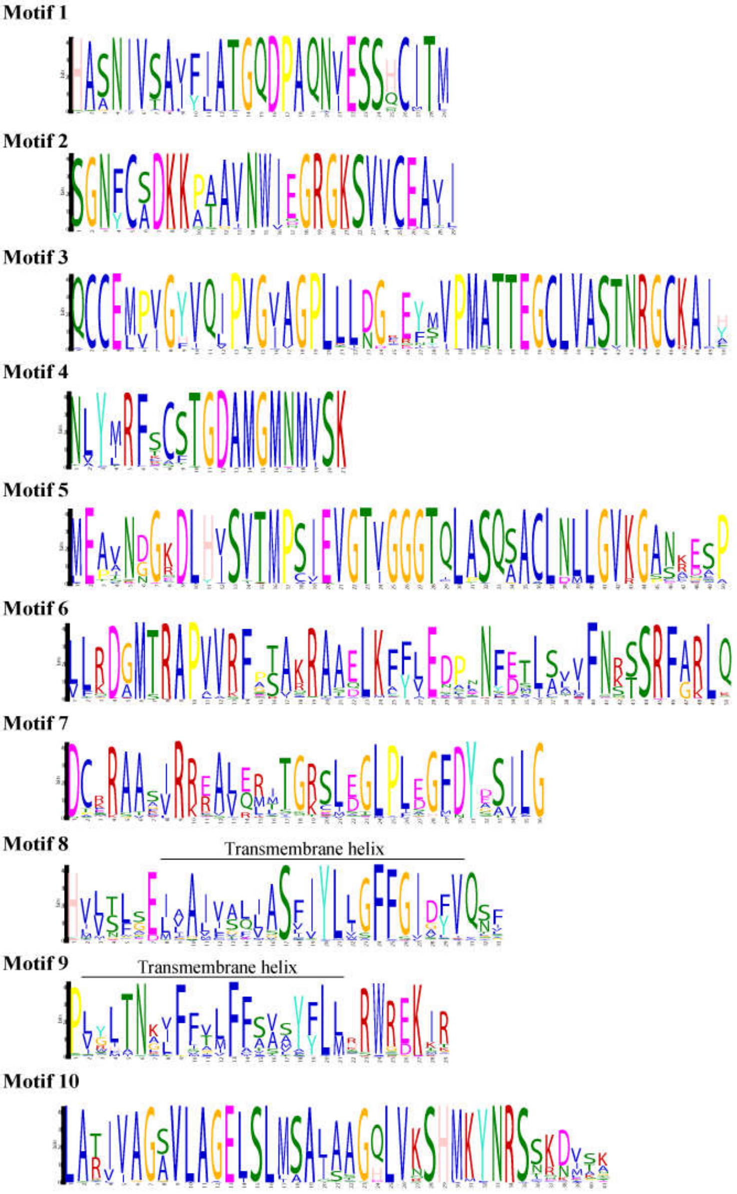

Figure S1 Sequence logos of 10 conserved motifs were identified in 124 HMGRs from 29 species using the MEME search tool.

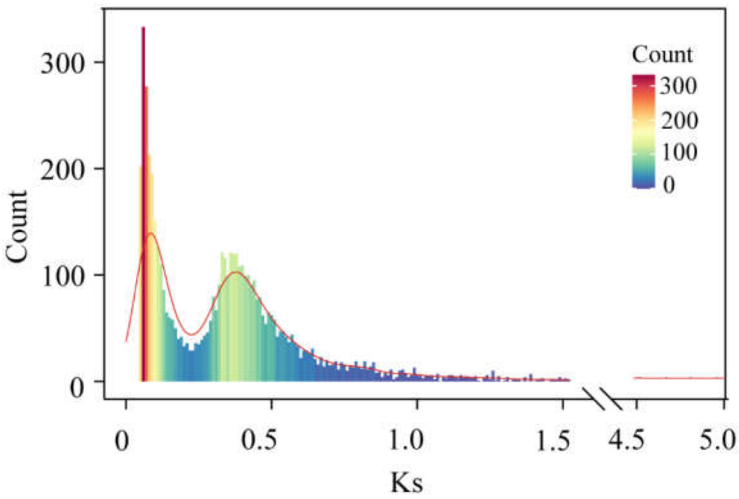

Figure S2 The Ks distributions of *L. erythrorhizon* synteny gene pairs.

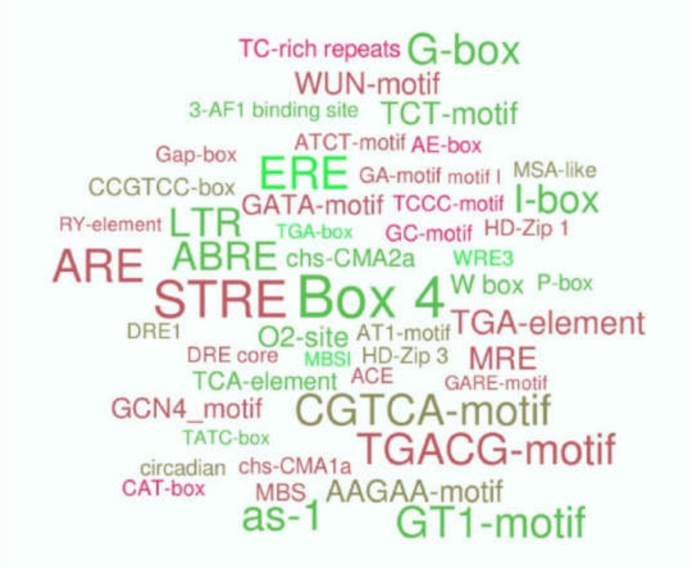

Figure S3 Cloud word graph of cis-acting elements in *LerHMGRs*'s promoters.

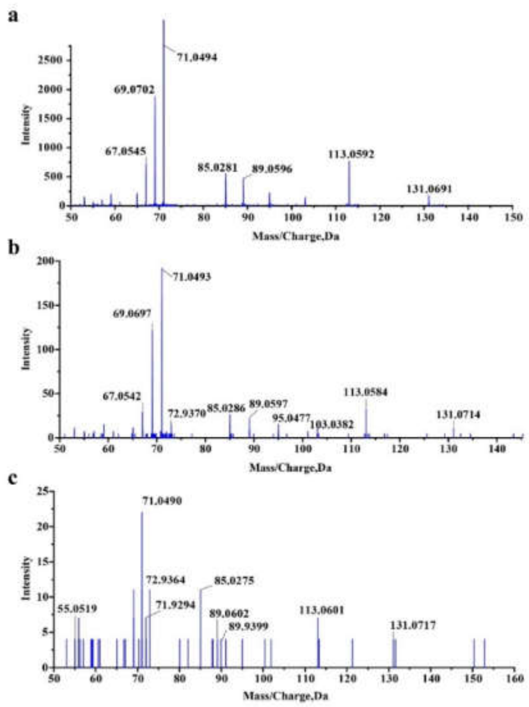

Figure S4 Secondary ion mass spectrometry of the enzymatic reaction productions were detected using UPLC.

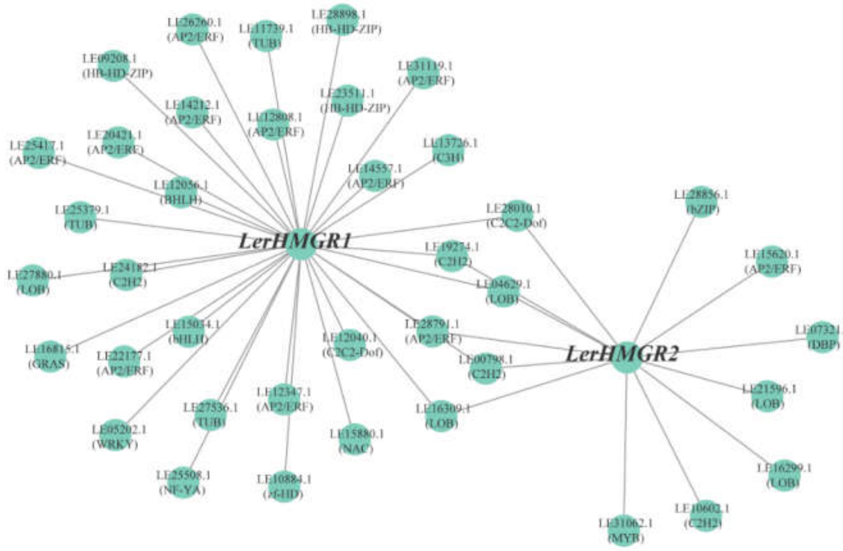

Figure S5 Co-expression network analysis of transcription factors with *LerHMGR1* and *LerHMGR2* in *L. erythrorhizon*.

The edges are drawn when the Pearson correlation coefficient is > 0.65 with p-value < 0.05.
